# Intra-Abdominal Desmoplastic Small Round Cell Tumor: Current Treatment Options and Perspectives

**DOI:** 10.3389/fonc.2021.705760

**Published:** 2021-09-15

**Authors:** Guixia Wei, Xinyao Shu, Yuwen Zhou, Xia Liu, Xiaorong Chen, Meng Qiu

**Affiliations:** ^1^Department of Abdominal Cancer, Cancer Center, West China Hospital of Sichuan University, Chengdu, China; ^2^Department of Biotherapy, Cancer Center, West China Hospital of Sichuan University, Chengdu, China

**Keywords:** intra-abdominal desmoplastic small round cell tumor, EWS-WT1 gene, treatment, targeted therapy, immunotherapy

## Abstract

Intra-abdominal desmoplastic small round cell tumor (IDSRCT) is a rare and highly malignant soft tissue neoplasm, which is characterized by rapid progression and poor prognosis. The mechanism underlying the development of this neoplasm remains elusive, but all cases are characterized by the chromosomal translocation t (11;22) (p13; q12), which results in a formation of EWSR1-WT1 gene fusion. The diagnosis of IDSRCT is often made with core-needle tissue biopsy specimens or laparoscopy or laparotomy. Immunohistochemical analyses have shown the co-expression of epithelial, neuronal, myogenic, and mesenchymal differentiation markers. FISH or reverse transcription polymerase chain reaction detecting EWS-WT1 fusion can be performed to assist in molecular confirmation. There is no standard of care for patients with IDSRCT currently, and majority of newly diagnosed patients received the aggressive therapy, which includes >90% resection of surgical debulking, high-dose alkylator-based chemotherapy, and radiotherapy. More recently, targeted therapy has been increasingly administered to recurrent IDSRCT patients and has been associated with improved survival in clinical conditions. Immunotherapy as a possible therapeutic strategy is being explored in patients with IDSRCT. In this review, we summarize currently available knowledge regarding the epidemiology, potential mechanisms, clinical manifestations, diagnosis, treatment, and prognosis of IDSRCT to assist oncologists in comprehensively recognizing and accurately treating this malignancy.

## 1. Introduction

**Figure 1:**
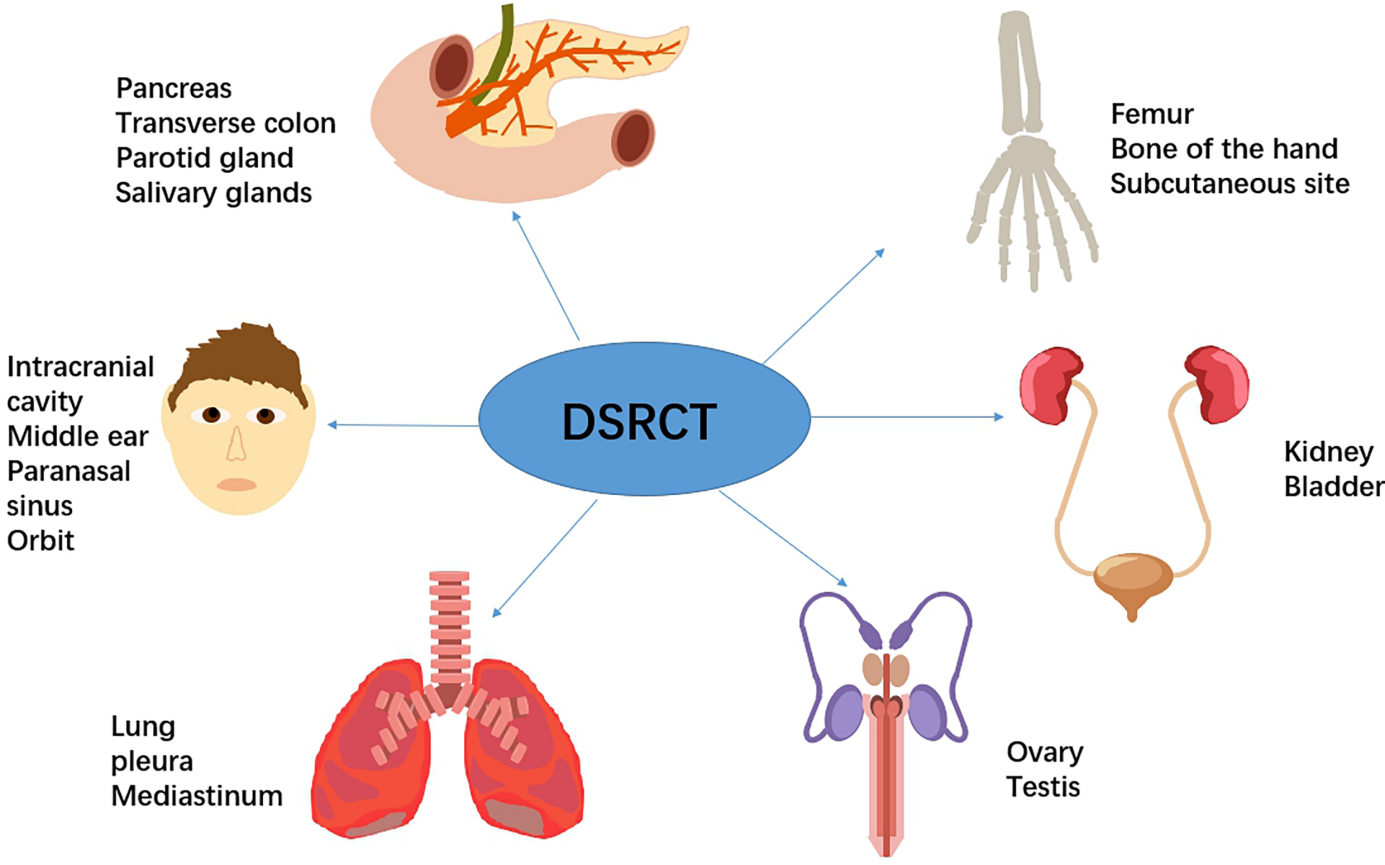
Previous case reports reported primary sites of DSRCT, including kidney (2), bladder (3), pancreas (4), transverse colon (5), testes (6), ovary (7), mediastinum (8), pleura (9), lung (10), parotid gland (11), salivary glands (12), middle ear (13), sinonasal (14), orbital (15), intracranial cavity (16), subcutaneous site (17), femur (18), and bone of the hand (19).

Desmoplastic small round cell tumor (DSRCT), according to the International Classification of Disease for Oncology (2020), is categorized as a malignant tumor of uncertain differentiation. DSRCT typically occurs in the abdominal cavity, which is known as intra-abdominal desmoplastic small round cell tumor (IDSRCT) (1). Other primary sites have been reported (**Figure 1**). It is a rare and aggressive malignant soft tissue sarcoma that predominantly occurs in young male adults (20). IDSRCTs are associated with chromosomal translocation t (11;22) (p13; q12), which results in a formation of EWSR1-WT1 gene fusion (21, 22). IDSRCT mainly originates in the abdominopelvic cavity, involving the mesentery and retroperitoneum (23, 24). There is a lack of international consensus regarding the treatment of IDSRCT, and therapeutic regimens were derived from Ewing sarcoma’s (ES) treatment because of the involvement of the EWS gene and activation of similar oncogenic pathways in both ES and IDSRCT. The prognosis of IDSRCT is poor, with a median 5-year survival rate of 15%–25% (25). Because of the rarity of this malignancy, very few studies have investigated it, and most of these studies are case reports. This review summarizes the current knowledge on the epidemiology, potential mechanisms, clinical manifestations, diagnosis, treatment, and prognosis of IDSRCT, highlighting the modalities of treatment.

## 2. Methods

This review summarizes the available literature on IDSRCT. We used the terms “abdominal,” “desmoplastic small round cell tumor,” and “intra-abdominal desmoplastic small round cell tumor” in our literature search. Previous reviews, articles, clinical trials, and case reports were included, and our search was not limited by language. Studies published from 1989 to 2021 were analyzed in the current review.

## 3. Epidemiology

IDSRCT is a rare and aggressive soft-tissue sarcoma. Its morbidity ranges between 0.2 and 0.74 cases per million people per year (26–28). IDSRCT mainly affects adolescents or young adults (median age at diagnosis: 27.0 years; range: 16–45 years), with a marked predominance in males (4:1 male-to-female ratio) (29, 30). No specific risk factors associated with the occurrence or progression of IDSRCT have been reported.

## 4. Mechanism

The pathogenesis of IDSRCT remains elusive. However, it is uniquely characterized by chromosomal translocation t (11;22) (p13; q12), which results in the fusion of the EWS and WT1 genes. The wild-type WT1 gene encodes a zinc finger-containing protein, which acts as a repressor of transcription. With the fusion of the EWS and WT1 genes, the normal function of the zinc finger region of the WT1 gene is lost, leading to the transcriptional activation of at least 35 downstream target genes (31, 32). These target genes encode growth factors and growth factor receptors, such as platelet-derived growth factor (PDGF)-α, Type-1 insulin-like growth factor receptor (IGF-1-R), and epidermal growth factor receptor (EGFR) (33), which are related to tissue differentiation and the proliferation, adhesion, and metastasis of the tumor cells (34).

Recent genomics analysis of mutational profiles indicated that epithelial–mesenchymal transition (EMT), immune response, and the DNA damage response (DDR) are associated with gene deregulation in DSRCT (35). Whole-exome sequencing of six consecutive pre-treated DSRCT samples identified 137 unique somatic mutations: 133 mutated genes were case-specific, and only 2 genes were overlapping among two cases but in different locations, which reveals the heterogeneity of the DSRCT genome. They also discovered that 27% of the 135 mutated genes are associated with DDR and EMT/mesenchymal–epithelial reverse transition (MErT), which could result in tumor extreme heterogeneity followed by genomic instability, and consequently produce drug resistance (35). MErT/EMT plays a crucial role in the metastasis and associated invasiveness of the sarcoma (36). Jiang et al. reported two novel somatic mutations, one associated with c-Met tyrosine kinase and the other related to PIK3CA gene, in 10 advanced stage DSRCT patients (37). The latter mutation was involved in the activation of the PI3K/AKT/mTOR pathway, facilitating the growth and proliferation of tumor cells (38).

## 5. Clinical Manifestations

**Figure 2:**
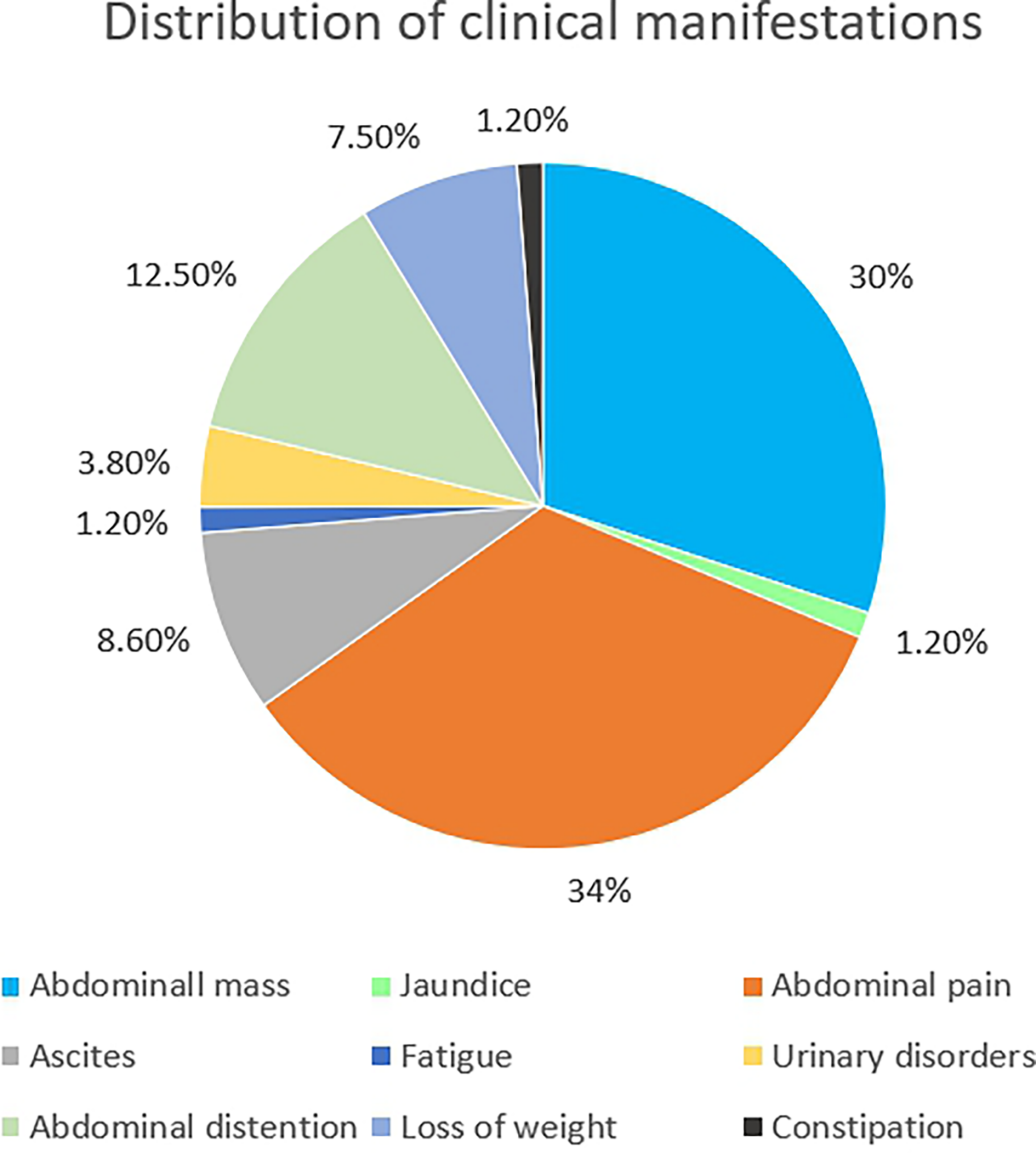
The features of clinical manifestation distribution of IDSRCT.

IDSRCT is commonly diagnosed at an advanced stage. Enlarged lymph nodes were seen in 50% of the patients, and distant metastasis was reported in 25% of the cases at the time of diagnosis (39). Widespread tumor nodules were also observed throughout the peritoneum, especially in the mesentery or omentum (40) (**Table 1**). The most common extraperitoneal metastasis is liver, followed by lymph node, bones, and lung (27, 28). The manifestations of IDSRCT are nonspecific and are related to the size, location, and speed of disease progression (40, 70, 71). It usually presents as a palpable abdominal mass with pain and other diverse symptoms, including distention, ascites, loss of weight, jaundice, fatigue, and constipation (**Figure 2**). Huge tumor masses can also cause compression symptoms, like intestinal obstruction and ureteral obstruction (72).

**Table 1:** Published case reports and case series regarding IDSRCT.

Reference	Patient	Diagnosis	Metastases	Treatment	Regimen	Response	PFS (m)	OS (m)
(41)	F/46	Resection biopsy	Omentum	C+S	HD-CAV	SD	1	NR
(42)	M/10	Resection biopsy	Liver, lung	C+R	VI	PR	46	50
(4)	M/9	Resection biopsy	NR	S+C	VAI	NR	7	NR
(43)	F/23	Biopsy	Liver lymph nodes	C	VDC	PD	NR	4
(44)	M/26	Biopsy	Pelvic	C+S+H+R	VDC/IE	CR	48	NR
(45)	M/18	Fine-needle aspiration	NR	C+S+T	Trabectedin	PR	8	48
(46)	M/14	Biopsy	Liver, spleen, kidney, pancreas	C+S+R	VDC/IE	SD	NR	NR
(47)	M/14	Resection biopsy	Liver	S+C	NR	Improved	NR	NR
(48)	M/27	Biopsy	Spleen, lymph node	C+S	VDC/IE	Improved	NR	NR
(49)	M/24	Resection biopsy	Lymph node, omentum	C+S	IMAP	Death	0	NR
(50)	M/21	Biopsy	Liver, Ileum, cecum	C+S	VDC/IE	SD	12	NR
(51)	M/39	Biopsy	Lymph node	C+S	VDC/IE	SD	NR	NR
(52)	M/16	NR	Prostate, rectum	C+S+R	VNR–CTX	PR	17	NR
(53)	M/26	Biopsy	Liver, lymph node	C	VNR–CTX	PR	4	NR
(53)	M/NR	NR	Bone, lymph nodes, liver	T	Sunitinib	SD	10	NR
(53)	M/NR	NR	Lymph nodes, peritoneum	T	Sunitinib	SD	4	NR
(53)	M/NR	NR	Peritoneum, lymph node	T	Sunitinib	SD	6	NR
(53)	M/NR	NR	Liver, peritoneum	T	Sunitinib	PD	1	NR
(53)	M/NR	NR	Liver, pleura, peritoneum	T	Sunitinib	PD	1	NR
(53)	M/NR	NR	Liver, lung, peritoneum	T	Sunitinib	PD	2	NR
(53)	M/NR	NR	Liver, peritoneum	T	Sunitinib	PR	10	NR
(53)	M/NR	NR	Peritoneum, lymph node	T	Sunitinib	PR	NR	NR
(54)	M/23	Biopsy	Liver, peritoneum	C+S	VDC/IE+ Trabectedin	SD	NR	24
(54)	M/19	NR	Liver, pleura, peritoneum	C	VDC/IE+ Trabectedin	SD	NR	16
(5)	F/30	Resection biopsy	No	S	No	SD	6	NR
(55)	M/31	Resection biopsy	Brain, liver, lymph nodes	S+C+R	NR	PD	NR	17
(56)	M/27	Tissue biopsy	Mesentery, peritoneum.	C	PAVEP	PR	NR	12
(57)	F/22	Frozen biopsy	NR	C	1.VDC2.VAC	PD	NR	9
(58)	M/18	Percutaneous biopsy	Mesentery, liver	C	VDC/IE +irinotecan	PR	NR	20
(59)	NR/NR	NR	NO	C+T	Pazopanib+ sirolimus	SD	NR	NR
(60)	F/11	Resection biopsy	Ovary	S+C	VDC/IE	PD	NR	11
(61)	F/7	Resection biopsy	NO	S+C	VDIE+VP-16	SD	NR	NR
(62)	M/38	Biopsy	Vessels, colon lymph nodes	S+R+C	CAP	CR	30	NR
(63)	F/30	Frozen biopsy	Ovary, lung, lymph node	S+C	VDC	PR	NR	NR
(64)	M/52	Biopsy	Omentum	C	VDC/IE	Death	NR	NR
(65)	M/16	Biopsy	Colon, stomach, spleen	S	NO	SD	NR	NR
(66)	M/33	Biopsy	NR	C	ICE	SD	NR	NR
(30)	M/11	Biopsy	NR	C+S	VAC/IVA	PR	NR	NR
(30)	M/7	Resection, biopsy	Omentum, bowel, pelvis	S+C	IRS-38	SD	18	NR
(30)	F/13	Biopsy	Small bowel	C+S+SCT	IRS-38	NR	9	11
(30)	M/11.5	Resection biopsy	Liver, bladder, colon	S+C	G-FLIP	NR	8	NR
(67)	F/26	Biopsy	Pancreas, vein, duodenum	C	VDC/IE	SD	NR	9
(68)	M/22	Resection biopsy	Liver, peritoneum	C	DC	PR	NR	13
(69)	M/29	Resection biopsy	Small bowel, lymph node	S+C+R	VDC/IE	Improved	NR	NR

IDSRCT, intra-abdominal desmoplastic small round cell tumor; C, chemotherapy; S, surgery; R, radiotherapy; T, target therapy; H, HIPEC; SD, stable disease; CR, complete response; PR, partial response; PD, progressive disease; M, male; F, female; EVAIA, etoposide, vincristine, doxorubicin, ifosfamide, and dactinomycin; VAC/IE, vincristine, actinomycin D, and cyclophosphamide, then alternated with ifosfamide and etoposide; IMAP, vincristine, doxorubicin, ciclofosfamida, isofosfamida, etoposide; VAI, ifosfamide, doxorubicin, vincristine; VDC, vincristine, doxorubicin, cyclophosphamide; PAVEP, cyclophosphamide, etoposide, doxorubicin, cisplatin; VI, irinotecan, vincristine; IT, irinotecan, temozolomide; PC, carboplatin, paclitaxel; DC, doxorubicin, cisplatin; VDIE, vincristine, doxorubicin, etoposide, ifosfamide; CAP, cyclophosphamide, adriamycin, cisplatin; ICE, IFM, etoposide, carboplatin; IVA, actinomycin-D, vincristine, ifosfamide; IRS-38, oncovin, platinol, adriamycin, cyclophosphamide; G-FLIP, Gemzar, 5-FU/leucovorin, camptothecin, platinol; SCT, blood stem cell transplantation; NR, not reported.

## 6. Diagnosis

The diagnosis of IDSRCT is often made with core-needle tissue biopsy specimens or laparoscopy or laparotomy (73). IDSRCT is thought to originate from progenitor cells with polyphenotypic potential (38). Macroscopically, IDSRCT is visible as a pale and firm mass in the peritoneal cavity with or without hemorrhage, necrosis, and cystic degeneration (20). Microscopically, histological examination shows uniform small round tumor cells grouped as clumps and nests with unclear cell borders, hyperchromatic nuclei, and inconspicuous nucleoli surrounded by hypocellular desmoplastic stroma. Glandular or rosette patterns or nuclear molding can be discovered. In addition, mitosis and apoptosis are common (71, 74, 75). However, a recent case report described a 12-year-old boy of IDSRCT characterized by spindle cells with scant cytoplasm and with no desmoplastic stromal reaction, which reveals the heterogeneity of morphologic features in IDSRCT (72). Immunohistochemical analyses of IDSRCT have shown the co-expression of epithelial (cytokeratin and epithelial membrane antigen), neuronal (NSE and CD56), myogenic (desmin), and mesenchymal (vimentin) differentiation markers and WT1 (C-terminus antibody) (**Table 2**). Notably, >90% of patients with DSRCT express desmin and EMA (80). Most IDSRCT patients typically do not express CD 99, HMB-45, S-100, and myogenin (75, 81)

**Table 2:** The features of immunohistochemical staining of IDSRCT reviewed from published case reports and case series.

	Vimentin	Desmin	EMA	CK	WT-1	CD99	Myogenin	S100	NSE	Leu-7
**Shen et al. (** **46** **)**	NR	+	NR	+	+	–	–	–	NR	NR
**Shi et al. (** **76** **)**	+	+	+	+	+	+	NR	–	+	NR
**Ambar et al. (** **42** **)**	NR	+	+	+	+	–	NR	NR	NR	NR
**Li et al. (** **24** **)**	NR	+	+/-	+/-	–	NR	NR	–	–	NR
**Reisner et al. (** **48** **)**	NR	+	NR	+	NR	+	NR	+	NR	NR
**Briseño-Hernández et al. (** **49** **)**	NR	+	+	NR	NR	NR	NR	NR	+	NR
**Kandhari et al. (** **50** **)**	+	+	NR	+	NR	NR	NR	NR	NR	NR
**Nathan et al. (** **51** **)**	NR	+	+	+	NR	NR	NR	+	+	NR
**Frezza et al. (** **54** **)**	NR	+	NR	+	NR	NR	NR	–	NR	NR
**Frezza et al. (** **110** **)**	NR	+	NR	–	–	NR	NR	–	–	NR
**Saleh et al. (** **4** **)**	NR	NR	+	NR	NR	+	–	NR	NR	NR
**Huang et al. (** **5** **)**	+	+	NR	+	+	NR	NR	NR	+	NR
**Ordi (** **77** **)**	+	+	+	+	NR	+	NR	NR	+	NR
**Takahira et al. (** **56** **)**	+	+	NR	+	NR	NR	NR	+	+	NR
**Wakahashi et al. (** **57** **)**	+	+	+	–	NR	NR	NR	–	NR	NR
**Hirano et al. (** **58** **)**	+	+	+	+	+	NR	NR	NR	NR	NR
**Slomovitz et al. (** **60** **)**	NR	+	+	+	NR	NR	NR	–	NR	NR
**Eaton et al. (** **61** **)**	NR	+	+	NR	+	+	–	NR	NR	NR
**Zhang et al. (** **62** **)**	NR	+	+	–	NR	–	NR	NR	NR	NR
**Ferreira et al. (** **44** **)**	NR	+	+	NR	+	NR	NR	NR	NR	NR
**Shimazaki (** **78** **)**	NR	+	+	+	NR	+	NR	–	+	NR
**Kim et al. (** **67** **)**	+	–	NR	–	NR	+	NR	NR	+	NR
**Devaney (** **79** **)**	+	+	+	+	NR	NR	NR	NR	NR	NR
**Ujihara et al. (** **66** **)**	NR	+	NR	+	–	NR	NR	–	NR	NR
**Takekawa et al. (** **65** **)**	+	+	+	NR	+	NR	NR	NR	+	NR
**Baz et al. (** **64** **)**	+	+	+	NR	+	–	NR	NR	NR	NR
**Xie et al. (** **63** **)**	+	+	+	NR	NR	–	NR	NR	NR	NR

IDSRCT, intra-abdominal desmoplastic small round cell tumor; +, positive; -, negative; +/-, suspicious positive; NR, not reported; EMA, epithelial membrane antigen; CK, cytokeratin; NSE, neuron-specific enolase.

As for molecular characteristics of DSRCT, previous literature described that almost all DSRCTs exhibit nuclear positivity for the DNA binding domain (C-terminal portion) of WT1, which can distinguish DSRCT from Wilms tumor and Ewing sarcoma (80). The specific chromosomal translocation t(11;22) (p13; q12) of DSRCT produces a chimeric EWSR1-WT1 fusion gene that encodes an aberrant transcription regulatory factor consisting of the C-terminal portion of WT1 and the trans-activation domain (N-terminal portion) of EWS (72). The EWSR1-WT1 fusion gene generally consists of exon 7 of the EWSR1 gene on chromosome 22 and exons 8–10 of the WT1 gene on chromosome 11 (82). However, several studies reported various alternative breakpoints for the t (11;22) (p13; q12) translocation (72, 83–85). Those fusion gene variants generally contain additional exons from EWS with conservation of the WT1 complement (EWS-WT1 8/8, 9/8, 10/8, and 9/7). Due to the heterogeneity of immunohistochemical findings, molecular methods are essential to verify diagnosis of IDSRCT (86). Molecular analysis by fluorescence *in situ* hybridization (FISH) and reverse transcription polymerase chain reaction to detect EWSR1 rearrangement and the EWSR1-WT1 fusion gene, respectively, has also been used to confirm IDSRCT diagnosis along with clinical findings (86). The molecular methods have higher sensitivity than that of immunohistochemical tools (87).

No specific tumor markers have been identified for the diagnosis of IDSRCT, except elevated serum CA125 and NSE levels in some patients (3, 57, 71). For imaging information, ultrasound usually shows lobulated peritoneal masses with variable echogenicity, and dystrophic intratumoral calcification is discovered in 20% of cases (75). Computed tomography (CT) is considered as a primary auxiliary method for the assessment of response and follow-up of IDSRCT, which typically show multifocal masses originating from the retroperitoneum or abdominopelvic cavity with poorly defined boundaries and unevenly enhanced signals. Cystic changes in large masses with heterogeneous enhancement can be found after contrast. Some cases had evidence of punctate calcification in primary mass (34, 88, 89). Twenty percent of IDRCT patients show ascites, and lymph node involvement can be seen in 50% of cases (75). Magnetic resonance imaging (MRI) can detect potential lesions that are not observed by CT alone. Due to the presence of necrosis, hemorrhage, and fibrous stroma in IDSRCT, it often shows heterogeneous high-intensity signals on T2-weighted images and hypointense or isointense signals on T1-weighted images in MRI with heterogeneous enhancement after gadolinium (74, 75). Although CT and MRI can help to identify primary sites of IDSRCT, they have limited power to show the metabolic activity of tumors, which promotes the application of 18F-fluorodeoxyglucose (FDG) positron emission tomography (PET) and CT (FDG PET/CT) in IDSRCT (90). A retrospective study indicated that FDG-PET may earlier predict histologic response to chemotherapy than macroscopic size change detected by CT. Although most patients obtain rapid symptom relief when treated with chemotherapy, the change of tumor size is generally minimal because of the presence of abundant stromal component in IDSRCT, while the great decrease of metabolic activity can be detected by FDG-PET earlier (91). Furthermore, the specificity of FDG PET/CT is as high as 97.4% in DSRCT lesions, especially for involved lymph node and bone metastatic lesions, allowing early discovery of recurrent IDRCT and change of treatment strategy (90, 92, 93). Thus, FDG PET-CT should be considered as the preferred imaging method for monitoring patients with IDSRCT (90).

## 7. Staging

There is no definite staging system for IDSRCT. Green et al. (94) proposed a staging system based on primary tumor burden, liver metastasis, and extra-abdominal metastasis. Primary tumor burden was measured using the peritoneal cancer index (PCI) score, calculated by measuring the tumor diameter in 13 abdominopelvic regions (95). In this staging system, a lower PCI score (<12) without liver or extra-abdominal metastasis was defined as Stage I; a higher PCI score (≥12) without liver or extra-abdominal metastasis was defined as Stage II; liver metastasis without extra-abdominal metastasis was defined as Stage III; and extra-abdominal metastasis regardless of PCI score was defined as Stage IV (**Table 3**). However, this staging system requires further validation before being applied to all DSRCT types.

**Table 3:** DSRCT staging criteria proposed by Hayes-Jordan, Green et.al.

Stage	Primary tumor (PCI)	Liver metastasis	Extra-abdominal metastasis
I	<12	No	No
II	≥12	No	No
III	Any	Yes	No
IV	Any	Yes or no	Yes

DSRCT, desmoplastic small round cell tumor; PCI, peritoneal cancer index.

## 8. Treatments

The therapeutic regimen for IDSRCT is mainly derived from ES treatment because of the similarity in the oncogenic pathways involved and EWS gene fusion in both malignancies (31). Although multimodal treatments have been proposed for IDSRCT patients, the prognosis remains poor (96). With the widespread utilization of targeted therapy, the prognosis of IDSRCT patients has markedly improved. Published case reports and case series of treatments administered to IDSRCT patients are summarized in **Table 1**.

### 8.1. Surgery

Radical surgical excision without residual disease is usually impossible because of the presence of multiple serosal tumor nodules and obscure boundary of IDSRCT (97). Thus, cytoreductive surgery (CRS) is regarded as the fundamental therapy for IDSRCT patients, which is defined as the resection of ≥90% of the tumor burden, preserving the non-invaded peritoneum macroscopically (70, 98). The lesions are generally confined to the serosal or superficial muscle layers, although there is dissemination throughout the peritoneal cavity, allowing the treatment of local tangential resection (99). The surgical resection extension is usually extensive, including the resection of primary disease with acceptable margins, peritonectomy, lymphadenectomy, and the resection of involved adjacent tissue (89). To preserve bowel length, wedge excision can be performed to remove masses that invade deeply into the bowel (99). According to the recent studies, patients treated with complete CRS have significantly improved survival compared with patients receiving insufficient or no surgery with macroscopic residual tumor (the 3-year survival rates 49.6% *vs.* 31.1% *vs.* 13.7%, p =  0.009) (70, 100). Complete resection of metastases combined with cytoreduction is essential in patients with extra-abdominal disease, while locoregional surgery alone typically cannot be considered if patients have extra-abdominal lesion. Moreover, it is not a first option for IDSRCT patients with extensive subdiaphragmatic lesion or unresectable periportal disease or widely infiltration or metastases of liver to received CRS (99). Based on the Sugarbaker completeness of cytoreduction (CR) score, the completeness of CRS was classified under four categories: CR 0, no macroscopically residual disease; CR 1, residual nodules smaller than 2.5 mm; CR2, residual nodule size ranging from 2.5 mm to 2.5 cm; and CR 3, residual nodule size larger than 2.5 cm (101). To achieve complete CRS, multivisceral resection is often needed, followed by various complications. Fifty-six percent of patients after surgery have mild complications, including urinary retention/urinary tract infection, wound infection, and ileus, while pelvic abscess, AKI, respiratory failure, and anastomotic dehiscence were reported in the residual 44% of patients who received surgery (102). Therefore, extensive surgical resection-associated complications should be considered before the administration of surgery.

### 8.2. Chemotherapy

IDSRCT is sensitive to chemotherapy; therefore, neoadjuvant chemotherapy is usually recommended for patients with advanced-stage and unresectable IDSRCT (27). Several studies have suggested that IDSRCT patients who have evident efficacy to neoadjuvant chemotherapy have an improved overall survival (OS) than those who are resistant to neoadjuvant therapy (27, 103). However, IDSRCT treated with neoadjuvant chemotherapy may not have a significant reduction of size because of its large stromal component, which should not delay or prevent an attempt of administration of CRS (91). The chemotherapy regimen of IDSRCT mainly follows the same schedule as that used in ES (25). The P6 chemotherapy regimen, comprising vincristine/doxorubicin/cyclophosphamide alternating with etoposide/ifosfamide (VDC/IE), is the most common neoadjuvant chemotherapy regimen for IDSRCT patients (43, 69). Vincristine, doxorubicin, and ifosfamide are a reasonable alternative regimen for older people who may not tolerate the intense regimen (73). For P6 chemotherapy-resistant patients, temozolomide/irinotecan, cyclophosphamide/topotecan, gemcitabine/docetaxel, and high-dose ifosfamide can be considered as second- or third-line chemotherapy regimens (**Table 4**) (75, 80, 104). Some IDSRCT patients receive adjuvant chemotherapy (high-dose 5-FU, temozolomide/irinotecan-based therapy, and high-dose ifosfamide) (104–106) in combination with radiotherapy after CRS to increase the effectiveness of the surgery (105, 106). Palliative chemotherapy is administered to patients with metastatic tumors at the time of diagnosis (27). Several regimens, such as irinotecan in combination with vincristine (42), vinorelbine plus low-dose cyclophosphamide (52), and trabectedin (45, 54), have been reported to show clinical benefits for refractory IDSRCT patients. Specifically, trabectedin has been shown to interact with the minor groove of DNA, affecting several transcription factors, DNA repair molecules, and DNA-binding proteins; perturbing the cell cycle; and subsequently causing the death of cancer cells (107). Trabectedin has been deemed safe and effective in pre-treated IDSRCT patients who were refractory to conventional chemotherapy and resection (45, 54).

**Table 4:** The common chemotherapeutic regimens for the treatment of DSRCT.

Regimen	Agent	Dose
**First-line**		
VDC/IE	Vincristine	1.5 mg/m^2^
	Doxorubicin	37.5 mg/m^2^
	Cyclophosphamide	1,200 mg/m^2^
	Etoposide	100 mg/m^2^
	Ifosfamide	1,800 mg/m^2^
VDI	Vincristine	1.5 mg/m^2^
	Doxorubicin	37.5 mg/m^2^
	Ifosfamide	1,800 mg/m^2^
VDIE	Vincristine	1.5 mg/m^2^
PAVEP	Doxorubicin	20 mg/m^2^
	Ifosfamide	3,000 mg/m^2^
	Etoposide	150 mg/m^2^
	Cyclophosphamide	300 mg/m^2^
	Etoposide	75 mg/m^2^
	Doxorubicin	40 mg/m^2^
	Cisplatin	100 mg/m^2^
**Second-line**		
	Temozolomide/irinotecan	NR
	Cyclophosphamide/topotecan	NR
GD	Gemcitabine	1,000 mg/m^2^
	Docetaxel	100 mg/m^2^
	High-dose ifosfamide	NR
VIP	Etoposide	NR
	Ifosfamide	NR
	Cisplatin	NR
**HIPEC**		
	Cisplatin	100 mg/m^2^
	Oxaliplatin	300–460 mg/m^2^
MC	Mitomycin	75 mg/m^2^
	Cisplatin	120 mg

DSRCT, desmoplastic small round cell tumor; NR, not reported.

Other special chemotherapy forms, such as hyperthermic intraperitoneal chemotherapy (HIPEC), are also considered in patients with extensive metastasis over the abdominal cavity (70). However, the role of HIPEC remains controversial. A previous retrospective study has reported a significant improvement in the 3-year OS of patients treated with surgery and HIPEC (94), and other studies have shown that CRS followed by HIPEC may improve the disease control rate (DCR) in patients with peritoneal surface metastasis (106). Conversely, some studies have suggested that HIPEC is not associated with a better outcome (27, 70). Few patients who received CRS and HIPEC have been reported to need long-term parenteral nutrition or surgical intervention due to severe side effects, such as hemorrhagic cystitis, adhesive bowel obstruction, and sclerosing peritonitis (108). Therefore, assessing the actual effectiveness of HIPEC requires further studies.

### 8.3. Radiotherapy

Because of the multicentric growth tendency in the abdominopelvic cavity of IDRCT, whole-abdominopelvic radiotherapy (WAP RT) is a more effective treatment than locoregional radiotherapy (93). WAP RT is occasionally used as a consolidative therapy after CRS to remove microscopic or minimal residual disease (<2 cm) (88, 99). Treatment is performed with megavoltage photon beams by AP/PA fields into the entire peritoneal cavity. The median dose for patients without residual disease is 30 Gy in 1.5–1.55 Gy fractions, while for patients with residual lesions, the dose can be increased to 45–50 Gy (70, 99). IDSRCT patients treated with WAP RT combined with CRS and chemotherapy experienced prolonged survival (104). As for complications, gastrointestinal and hematological toxicities were the most common reported acute complications, while small bowel obstruction was the most common late toxicity in IDSRCT patients who underwent WAP RT (109). Acute complications can be treated with supportive care or symptomatic treatment, while surgical intervention is required in up to 10% of small bowel obstruction (106). Patients subjected to intensity-modulated radiation therapy (IMRT) reported a lower incidence of hematological toxicities compared to those who underwent WAP-RT (109). IMRT was found to selectively decrease the irradiated doses of adjacent normal organs (e.g., liver, kidneys, and pelvis), allowing even dose distribution to the peritoneal surfaces (106). Furthermore, there is no significant difference in OS between patients treated with IMRT and WART (109). In addition to being used as an adjunctive treatment, radiation can also be administered as a palliative treatment for recurrent IDSRCT (104).

### 8.4. Targeted Therapy

The formation of EWSR1-WT1 fusion gene can activate downstream signaling pathways, including platelet-derived growth factor (PDGF), vascular endothelial growth factor (VEGF), and insulin growth factor (IGF)-1. Those pathways may be potential targets for treatment of IDSRCT (38). Until now, targeted therapy has shown its clinical benefit in IDSRCT patients who had tumor relapse or progression despite first-line or second-line treatment (38). Based on previous studies, tyrosine kinase inhibitors (TKI), including pazopanib (110), sunitinib (53), sorafenib (23), anlotinib (104), apatinib (76), imatinib (111), anti-VEGFR monoclonal antibody (e.g., bevacizumab) (68), IGF-1-R inhibitors (112, 113), mTOR inhibitors (114), and PARP inhibitors (100) were used for the therapies and were found to be effective in select IDSRCT cases. Targeted therapy-treated patients who were reviewed from case reports/series and our hospital are listed in **Table 5**. All ongoing clinical trials are summarized in **Table 6**.

**Table 5:** Retrospectivec cases from published reports and our hospital of IDSRCT patients treated with target therapy.

Reference	Patient	1-line therapy	2-line therapy	3-line therapy	4-line therapy	5-line therapy	6-line therapy	Response	T-PFS(m)	OS(m)
(115)	31/M	Gemcitabine+ nedaplatin	Cyclophosphamide-epirubicin-vincristine **Apatinib**	NA	NA	NA	NA	PR	NR	NR
(76)	32/M	**Apatinib**	NA	NA	NA	NA	NA	Improved	NR	NR
(68)	37/M	PCB+ **bevacizumab**	NA	NA	NA	NA	NA	PR	NR	34
(23)	NR	MAID-ASCT	Gemcitabine-cisplatin	**Sunitinib**	NA	NA	NA	NR	2	NR
(23)	NR	VAI-Adriamycin- cisplatin- etoposide-cyclophosphamide	Carboplatin-etoposide	**Sunitinib**	NA	NA	NA	NR	5.5	NR
(23)	NR	Cyclophosphamide-etoposide- carboplatin	Etoposide-carboplatin-busulfan-thiotepa	Temozolomide	Temozolomide-Irinotecan	**Bevacizumab**	NA	Death	2	NR
(23)	NR	MAI	VAC	Ridaforolimus **Sunitinib**	NA	NA	NA	NR	4	38
(23)	NR	IVADo **Bevacizumab**	Navelbine-cyclophosphamide	Dalotuzumab **Sunitinib**	NA	NA	NA	NR	3	NR
(23)	NR	Adriamycin-ifosfamide-etoposide	Cyclophosphamide	Trabectedin	**Sunitinib**	NA	NA	NR	2	19
(23)	NR	MAI	VAC	**Sunitinib**	NA	NA	NA	NR	2	NR
(23)	NR	LV5Fu-Ciplatine	HIPEC-FOLFIRI	FOLFIRI	Holoxan-etoposide	AC	**Sorafenib**	PD	3.5	NR
(23)	NR	MAI	Gemcitabine-docetaxel	AI	Cisplatin-irinotecan	Trabectedin	**Sorafenib**	PD	4	NR
(116)	38/M	Chemotherapy (NR)	**Anlotinib**	NA	NA	NA	NA	NR	4	NR
Hospital	46/M	MAID	GD	**Apatinib**	NA	NA	NA	PR	>14	>21
Hospital	28/M	VAI, **Anlotinib**	NA	NA	NA	NA	NA	SD	>6	NO

IDSRCT, intra-abdominal desmoplastic small round cell tumor; M, male; F, female; T, target therapy; PR, partial response; OS, overall survival; PFS, progression-free survival; ASCT, autologous stem cell transplantation; NR, not reported; NA, not available; PCB, carboplatin-paclitaxel; MAID, mesna-adriblastine-ifosfamide-dacarbazine; ASCT, autologous stem cell transplantation; HIPEC, hyperthermic intraperitoneal chemotherapy; FOLFIRI, 5 FU-oxaliplatin-irinotecan; MAI, mesna-adriblastine-ifosfamide; VAI, vincristine-actinomycin-ifosfamide; VAC, vincristine-actinomycin D-cyclophosphamide; IVADo, ifosfamide-vincristine-actinomycin D-doxorubicin; GD, gemcitabine plus docetaxel; AC, adriamycin-cyclophosphamide; AI, adriamycin-holoxan. The bold values represent the targeted therapy during the treatment process.

**Table 6:** Clinical trials of DSRCT patients.

ClinicalTrials.gov Identifier	Conditions	Status	Phase	Interventions	Comparison	Participants
***Chemotherapy***						
NCT04095221	Relapsed/refractory DSRCT, rhabdomyosarcoma	Recruiting	I/II	Prexasertib + Irinotecan	Single-arm	30
NCT03275818	Relapsed/refractory sarcoma, including DSRCT	Active	II	Nab-paclitaxel	Single-arm	60
NCT00055952	Relapsed or refractory DSRCT, ES, PNET	Completed	II	Exatecan mesylate	Single-arm	NR
***Radiotherapy***						
NCT01277744	DSRCT patients underwent surgery	Completed	II	HIPEC with cisplatin	Single-arm	22
***Target therapy***						
NCT01189643	Newly diagnosed DSRCT	Active	I	Irinotecan + Temozolomide + Bevacizumab + High Dose Alkylator-Based Chemotherapy	Single-arm	15
NCT00417807	Refractory DSRCT	Completed	I/II	Imatinib mesylate	Single-arm	9
NCT00563680	Relapsed/refractory DSRCT, ES	Completed	II	AMG 479 (anti-IGF-1R)	Single-arm	38
NCT00062205	Relapsed/refractory DSRCT, ES	Completed	II	Imatinib mesylate	Single-arm	40
***Target therapy+ Chemotherapy***						
NCT00720174	Unresectable/metastatic soft tissue sarcoma, including DSRCT	Completed	I	Cixutumumab and doxorubicin hydrochloride	Single-arm	30
NCT04145349	Refractory/relapsed DSRCT	Recruiting	I/II	Ramucirumab + Cyclophosphamide + Vinorelbine	Cyclophosphamide + Vinorelbine	34
NCT01946529	DSRCT, ES, PNET	Active	II	VDC/IE + temsirolimus/sorafenib/temozolomide/bevacizumab	VDC/IE	24
***Immunotherapy***						
NCT02982941	B7-H3-expressing relapsed/refractory solid tumors, including DSRCT	Completed	I	Enoblituzumab	Single-arm	25
***Radioimmunotherapy***						
NCT01099644	Solid tumors Involving the peritoneum, including DSRCT	Active	I	Radioimmunotherapy with 131I-8H9	Single-arm	54
NCT04022213	Solid tumors involving the peritoneum, including DSRCT	Recruiting	II	131 I-omburtamab + radiation	131 I-omburtamab	55
***Other therapy***						
NCT04213794	Resectable, refractory/recurrent abdominopelvic tumors, including DSRCT	Recruiting	I	HIPEC with doxorubicin and cisplatin	Single-arm	43
NCT04530487	Recurrent/refractory solid tumors, including DSRCT	Recruiting	II	ASCT after chemotherapy	Single-arm	40

DSRCT, desmoplastic small round cell tumor; ES, Ewing’s sarcoma; PNET, primitive neuroectodermal tumor; WT, Wilms tumor HIPEC, hyperthermic intraperitoneal chemotherapy; VDC/IE, vincristine/doxorubicin/cyclophosphamide alternating with etoposide/ifosfamide, CHPP, continuous hyperthermic peritoneal perfusion; ASCT, allogeneic stem cell transplantation.

#### 8.4.1. Pazopanib

Pazopanib, a TKI, targets the stem cell factor receptor c-Kit, PDGFR-α and -β, and VEGFR-1, 2, and 3 to prohibit angiogenesis and tumor proliferation (117). Pazopanib has been approved by the U.S. Food and Drug Administration (FDA) and the European Medicines Agency for the treatment of patients with renal cell carcinoma (RCC) and sarcoma (118). IDSRCT overexpresses VEGF-A, VEGFR-2, and PDGF, which gives rationality to the administration of pazopanib in pretreated IDSRCT patients (119, 120). Pazopanib has been demonstrated to achieve a variable DCR of 61% and 78% for patients who received prior chemotherapy and those who did not, respectively. Median progression-free survival (PFS) of 9.2 months and OS of 15.4 months have also been observed with pazopanib treatment. The common adverse reactions following this treatment were neutropenia, hypertension, fatigue, and diarrhea (110, 121).

#### 8.4.2. Sunitinib

Sunitinib has a mechanism of action like that of pazopanib, and it targets PDGFR, VEGFR, c-KIT, RET, colony-stimulating factor 1, and Flt3 (122). The FDA has approved sunitinib for the management of imatinib-refractory gastrointestinal stromal tumors (GISTs), RCC, and pancreatic neuroendocrine tumor (123). This agent has also shown clinical effectiveness in pretreated IDSRCT patients. In a case series comprising eight patients, six pretreated IDSRCT patients achieved an improved clinical outcome (partial remission [PR] and stable disease [SD]) after sunitinib treatment, and no severe adverse response was reported (53).

#### 8.4.3. Sorafenib

Sorafenib, a multitargeted TKI, targets c-KIT, PDGFR-β, VEGFRs 2–3, and Flt-3. Sorafenib has been approved for treating hepatocellular carcinoma, RCC, and thyroid cancer (124). In a retrospective study, two progressive IDSRCT patients received sorafenib and progressed at 4 months. Of the two patients, one developed skin toxicity, while the other had to terminate the treatment due to severe abdominal pain (grade = 3) (23). The true efficacy of sorafenib treatment for DSRCT patients requires large sample data to further validate the results.

#### 8.4.4. Apatinib

Apatinib, a novel multitargeted TKI, inhibits the expression of VEGFR-2, RET, c-Kit, and c-Src tyrosine kinases, selectively targeting VEGFR-2 (125). Apatinib is used for treating several tumor types, including gastric neoplasm (126), non-small cell lung cancer (NSCLC) (127), and colorectal cancer (CRC) (128). A case report showed that the tumor-related symptoms of IDSRCT patients were reduced quickly after the initiation of apatinib treatment (76). In another case report, one patient with IDSRCT received systemic chemotherapy (cyclophosphamide, epirubicin, and vincristine) plus apatinib and achieved PR after two cycles of treatment (115). Apatinib may be an alternative strategy for patients with IDSRCT. In a previous study, we presented the case of a 46-year-old man with metastatic IDSRCT admitted to our hospital. Although he had failed the first-line (MAID: mesna, doxorubicin, ifosfamide, and dacarbazine) and second-line chemotherapies (GD: gemcitabine plus docetaxel), he showed positive response to apatinib and quickly reached PR and achieved more than 14 months of PFS. This patient needed to reduce the dose of apatinib because of grade 3 hypertension and grade 2 digestive tract reaction. These adverse reactions resolved rapidly after dose reduction and usage of hypotensors.

#### 8.4.5. Anlotinib

Anlotinib mainly targets VEGFRs 1–3, PDGFR-α and -β, fibroblast growth factor receptors (FGFRs) 1–4, EGFR, c-Kit, Met, and stem cell factor receptors (104, 116). Anlotinib plays a crucial role in the treatment of NSCLC and thyroid cancer (129). A case report showed that one IDSRCT patient who progressed after resection and chemotherapy (ifosfamide and liposomal doxorubicin) received anlotinib as the second-line therapy, which reduced invasive lymph node size with a PFS of 4 months. Only fatigue (grade = 1) and high triglyceride levels (grade = 1) were observed as side effects (104). In a previous study, we presented the case of a 28-year-old man admitted to our hospital with multiple metastatic and unresectable IDSRCT lesions. He received anlotinib plus chemotherapy (VAI) as the first-line treatment and achieved an SD of over 6 months.

#### 8.4.6. Imatinib

Imatinib mesylate has been recommended as a treatment for GISTs and chronic myeloid leukemia (130). Notably, this agent also targets other tyrosine kinase receptors, such as PDGF-R and c-KIT (111). PDGF-A and PDGFR-β are related to tumor cell growth and proliferation (38) and are overexpressed in DSRCT (119). However, the efficacy of imatinib for IDSRCT has been found to be limited. Eight patients who were refractory to conventional treatment were administered imatinib, and only one patient achieved SD, while seven patients progressed rapidly 3 months after the initiation of imatinib. The toxicities were tolerated, and no severe adverse events were observed (111). Similarly, a phase II study suggested that imatinib was not associated with clinical benefits in pediatric patients with refractory DSRCT (131).

#### 8.4.7. Monoclonal Antibody Targeting VEGF

Bevacizumab is a monoclonal antibody that targets VEGF-A. It has been recommended for the treatment of metastatic CRC, advanced NSCLC, advanced cervical cancer, and metastatic RCC (132). A previous study has found that VEGFR-2 and VEGFA are overexpressed in DSRCT cell lines and xenograft models (31). One IDSRCT patient who underwent prior partial resection received bevacizumab and achieved an OS of 34 months (68). Notably, bevacizumab in combination with WART can be used to eradicate residual disease after surgery because it increases the sensitivity of the tumor nodules to radiotherapy (106). An ongoing clinical trial (NCT01189643) is exploring the combination of irinotecan, temozolomide, and bevacizumab in combination with standard P6 regimen for treatment of newly diagnosed patients with DSRCT. Similarly, ramicirumab, an anti-VEGF-2 monoclonal antibody, in combination with chemotherapy, is being assessed in a phase II clinical trial on DSRCT (**Table 4**; NCT04145349).

#### 8.4.8. IGF1R Inhibitor

IGF1R inhibitors can inhibit the proliferation of tumor cells by blocking the binding between IGF1R and IGF-1 and 2 (133). In addition, these inhibitors exert anti-angiogenic effects by targeting VEGF (134). Common IGF1R inhibitors include ganitumab and cixutumumab. IGF1R inhibitors are used for treating ES (135). In a Phase II study, 25% of clinical benefit (DCR ≥ 6 months) was achieved in DSRCT patients treated with ganitumab. This study suggested that ganitumab could prolong the survival of DSRCT patients with a median PFS of 19.0 months. Notably, five patients had to undergo dose reductions due to thrombocytopenia (grade = 3) caused by ganitumab. Other grade 3 toxicities, including hyperglycemia, leukopenia, neutropenia, and grade 4 thrombocytopenia, were also reported (112). In another study, SD was observed in two-thirds of pretreated patients who received cixutumumab in combination with temsirolimus (an mTOR inhibitor) (113).

#### 8.4.9. mTOR Inhibitor

The PI3K/Akt/mTOR pathway is constitutively activated in DSRCT progression, mainly through mTORC-2 expression (136), and it is related to cancer cell growth and survival in sarcoma (137). One IDSRCT patient, who resisted chemotherapy and anti-androgen therapy, maintained an SD of 10 months when treated with temsirolimus (114). Furthermore, mTOR inhibitor plus pazopanib led to an SD of 11 months in one IDSRCT patient who progressed after treatment with pazopanib alone (59). Nevertheless, mTOR inhibitor plus IGF1R inhibitor showed limited efficacy in DSRCT treatment (138).

#### 8.4.10. PARP Inhibitors

The DDR network may be a potential target for DSRCT due to the recent discovery that 27% of mutated genes of DSRCT are related to DDR (35). Poly (ADP-ribose) polymerase 1 (PARP1) is a key enzyme for base excision repair of single-strand DNA breaks, participating the recruitment of DNA repair proteins (139). PARP inhibitor has been reported to be effective in sarcoma with deficiency in DDR (38). Anke E. M. van Erp and coworkers discovered that PARP1 expression was observed in 100% of clinically derived DSRCT tumor tissues (100). They also found that DSRCT cells have a high sensitivity profile to the PARP inhibitor olaparib, and the combination of olaparib with temozolomide (an alkylating agent) in JN-DSRCT-1 cells results in synergistic effects, inducing cell cycle arrest followed by cell apoptosis, consequently leading to tumor reduction *in vitro* and *in vivo*.

IDSRCT patients who progress or relapse after first- and second-line treatment can first receive anti-VEGF monoclonal antibodies, like bevacizumab or TKI, that act on multiple pathways simultaneously (e.g., c-Kit, PDGFR-α and -β, and VEGFR-1, 2, and 3, Flt3, RET), including pazopanib, sunitinib, anlotinib, and apatinib. Among them, imatinib and sorafenib caused no benefit, which should not be considered in IDSRCT patients. Subsequent lines of management include IGF1R inhibitor or mTOR inhibitor. Notably, the effect of PARP inhibitor needs to be further verified in clinical studies. The existing preclinical and clinical data suggest some effect of targeted therapy in IDSRCT patients. However, the observed role predominantly is tumor stabilization rather than disease regression. Those potential targets are worthy of further exploration (23, 53, 68, 76, 100, 110–114).

### 8.5. Immunotherapy

Although immunotherapy has shown an unprecedented response in multiple tumor types, there is no clinical evidence of its efficacy in IDSRCT. Some potential targets for immunotherapy have been detected in IDSRCT. CD276, now called B7H3, and GD2 are expressed in 96% and 70% of DSRCT patients, respectively (140). B7H3 can lead to tumor cell immune evasion by inhibiting the effect of T cells, allowing tumor growth and metastasis (33). A phase I study indicated that intraperitoneal radioimmunotherapy with 131I-omburtamab (an anti-B7H3 monoclonal antibody) is well tolerated in patients with DSRCT (141). In addition, anti-B7H3 CAR-T cell immunotherapy has been evaluated in a phase I study for recurrent/refractory solid tumors, including DSRCT (**Table 4**; NCT04483778; NCT02982941). A clinical trial is studying the efficacy of intraperitoneal radioimmunotherapy with 131I-8H9 for patients with DSRCT (NCT01099644). The function of GD2 in IDSRCT has not been adequately identified. However, some studies have indicated that GD2 might increase the adhesion between extracellular matrix proteins and tumor cells and contribute to the metastasis of the neoplasm (33). Notably, GD3, an upstream molecule in the biosynthesis of GD2, has also been found in 70.0% of DSRCT patients. We speculate that GD3 may be a potential target of immunotherapy in IDSRCT patients (142). Immune checkpoint inhibitors targeting PD-1 and CTLA-4 have been demonstrated to prolong survival in several tumors, including NSCLC, RCC, and melanoma (143), but its clinical effect in IDSRCT remains unknown. Pembrolizumab, an anti-PD-1 agent, is now being tested in patients with rare sarcoma (including 6 patients with DSRCT) in a phase 2 clinical trial (NCT03012620).

### 8.6. Androgen Blockade Agent

Bulbul et al. reported higher expression of androgen receptor in DSRCT patients than in those with ES (144). Hence, androgen blockade agents were tested in IDSRCT patients, but its efficacy remains controversial. In a case report analyzing six patients treated with combined androgen blockade, 50% of the patients showed a positive response to the therapy (132), while in one patient, the tumor progressed after 2 months of treatment (114).

### 8.7. Autologous Stem Cell Transplant

ASCT has been performed in IDSRCT patients (145). In a case report, a complete response (CR) was seen in IDSRCT patients after resection, chemotherapy (VAC+VAP), and ASCT (146). Another study also found that ASCT could significantly improve OS in DSRCT patients who were in remission (147). Moreover, a retrospective study reported improved disease-free survival and OS in ASCT-treated DSRCT patients with residual tumors (148). Owing to the small sample size of the abovementioned studies, the true effectiveness of ASCT needs further validation in the future.

## 9. Palliative Care and Complications Management

Advanced IDSRCT patients with extensive systemic metastases who cannot tolerate surgery or systemic chemotherapy may consider targeted therapy with mild adverse reactions, including TKI inhibitors, mTOR inhibitors, or bevacizumab (53, 59, 68, 121). Nutritional care is essential for patients with advanced tumors. Pharmacological agents and pharmaconutrients have limited effects in patients with advanced cancer. If possible, patients with advanced tumor should engage in regular physical activity and adopt a prudent diet (149). For patients with tumor mass-associated compression symptoms, like intestinal obstruction and ureteral obstruction, palliative surgery can be considered, but the benefits and risks of palliative surgery must be balanced with special consideration in patients with advanced IDSRCT.

## 10. Prognosis and Follow-Up

The prognosis of IDSRCT is poor. Patients with IDSRCT had a dismal survival with 3- and 5-year survival rates from 44% to 15%–25% and a median OS of 1725 months (70, 75). Patients with hepatic/portal metastasis (150), resistance to neoadjuvant chemotherapy (27), and CD99 staining positive expression (100) have worse prognosis. Furthermore, local solitary lesions, no metastases, complete CRS, and adjuvant chemotherapy were independent risk factors for OS of IDSRCT (151). Some studies argued that some factors, such as sex, age, postoperative complications, lymph node metastases, the presence of extra-abdominal lesions at initial presentation, and tumor size (1, 29, 102), have no significant effect on the survival of IDSRCT. Despite the implementation of multimodal therapy, most IDSRCT patients experience quick relapses with a median PFS of 10–14 months (27, 75). Therefore, close follow-up is necessary after completion of treatment. If possible, PET-CT scan every 3 to 6 months is required, which can detect the change of metabolic activity prior to macroscopic neoplasm growth (90).

## 11. Future Perspectives

CRS combined with VDC/IE chemotherapy regimen and WAP-RT are regarded as the standard treatments for IDSRCT. Other therapies, such as molecularly targeted treatments, ASCT, and androgen blockade agents, can be considered when tumor progresses or relapses after standard therapy. Despite multimodal treatments, IDSRCT still has a dismal clinical outcome. The targeted agents for optimal effectiveness for IDSRCT remain unclear. Further research should be directed at exploring the potential effect of various targeted therapies in these patients with IDSRCT. Several potential targets like the DDR or MErT/EMT are waiting further exploration. Furthermore, immunotherapy as a possible therapeutic choice has limited extent effect in preclinical studies, which should be further verified in clinical conditions in the future.

## 12. Conclusion

IDSRCT refers to a rare and aggressive soft tissue malignancy that predominantly occurs in a young male population. It is often diagnosed with extensive peritoneal metastasis and has a dismal prognosis. Immunohistochemical analyses of IDSRCT are characterized by co-expression of epithelial, neuronal, and mesenchymal differentiation markers. FISH or reverse transcription polymerase chain reaction detecting EWS-WT1 fusion can be performed to assist in molecular confirmation. Multimodal treatments, including surgery, chemotherapy, and radiotherapy, in patients with IDSRCT have achieved improved outcome. Currently, targeted therapy and immunotherapy have expanded the treatment options for IDSRCT patients and are associated with improved survival rates in preclinical or clinical studies. Hepatic/portal metastasis and effect of neoadjuvant chemotherapy, number of lesions, and surgery types are associated with prognosis of IDSRCT. Due to the rarity and poor prognosis of this neoplasm, further studies are required to propose effective regimens for IDSRCT patients.

## Data Availability Statement

The original contributions presented in the study are included in the article/supplementary material. Further inquiries can be directed to the corresponding author.

## Author Contributions

GW reviewed the literature, and wrote the manuscript. XS wrote and revised the manuscript. YZ rechecked the manuscript. XL and XC assisted in drawing. MQ designed and revised the manuscript.

## Funding

This work was supported by the research and development important project of the Science and Technology Bureau in Sichuan (2018SZ0188).

## Conflict of Interest

The authors declare that the research was conducted in the absence of any commercial or financial relationships that could be construed as a potential conflict of interest.

## Publisher’s Note

All claims expressed in this article are solely those of the authors and do not necessarily represent those of their affiliated organizations, or those of the publisher, the editors and the reviewers. Any product that may be evaluated in this article, or claim that may be made by its manufacturer, is not guaranteed or endorsed by the publisher.
